# Quantification and expert evaluation of evidence for chemopredictive biomarkers to personalize cancer treatment

**DOI:** 10.18632/oncotarget.13544

**Published:** 2016-11-24

**Authors:** Shruti Rao, Robert A. Beckman, Shahla Riazi, Cinthya S. Yabar, Simina M. Boca, John L. Marshall, Michael J. Pishvaian, Jonathan R. Brody, Subha Madhavan

**Affiliations:** ^1^ Innovation Center for Biomedical Informatics, Georgetown University Medical Center, Washington, D.C., USA; ^2^ Department of Oncology, Lombardi Comprehensive Cancer Center, Georgetown University Medical Center, Washington, D.C., USA; ^3^ Department of Biostatistics, Bioinformatics, and Biomathematics, Georgetown University Medical Center, Washington, D.C., USA; ^4^ Pancreas, Biliary and Related Cancer Center, Department of Surgery, Thomas Jefferson University, Philadelphia, PA, USA; ^5^ Department of Surgery, Albert Einstein Medical Center, Philadelphia, PA, USA; ^6^ Otto J. Ruesch Center for the Cure of Gastrointestinal Cancer, Lombardi Comprehensive Cancer Center, Georgetown University Medical Center, Washington, D.C., USA

**Keywords:** precision medicine, predictive biomarkers, clinical utility, biocuration, evidence framework

## Abstract

Predictive biomarkers have the potential to facilitate cancer precision medicine by guiding the optimal choice of therapies for patients. However, clinicians are faced with an enormous volume of often-contradictory evidence regarding the therapeutic context of chemopredictive biomarkers.

We extensively surveyed public literature to systematically review the predictive effect of 7 biomarkers claimed to predict response to various chemotherapy drugs: ERCC1-platinums, RRM1-gemcitabine, TYMS-5-fluorouracil/Capecitabine, TUBB3-taxanes, MGMT-temozolomide, TOP1-irinotecan/topotecan, and TOP2A-anthracyclines. We focused on studies that investigated changes in gene or protein expression as predictors of drug sensitivity or resistance. We considered an evidence framework that ranked studies from high level I evidence for randomized controlled trials to low level IV evidence for pre-clinical studies and patient case studies.

We found that further in-depth analysis will be required to explore methodological issues, inconsistencies between studies, and tumor specific effects present even within high evidence level studies. Some of these nuances will lend themselves to automation, others will require manual curation. However, the comprehensive cataloging and analysis of dispersed public data utilizing an evidence framework provides a high level perspective on clinical actionability of these protein biomarkers. This framework and perspective will ultimately facilitate clinical trial design as well as therapeutic decision-making for individual patients.

## INTRODUCTION

Rapid advances in cancer research have enabled the identification and characterization of driver mutations, expression changes and structural variations in genes that should translate into better selection of therapies for patients. Remarkable successes in this field include the FDA approval of Trastuzumab for breast cancer patients overexpressing the HER2 protein [1] and Erlotinib for metastatic non-small cell lung cancer patients (NSCLC) with exon 19 deletion or exon 21 (L858R) substitution in the *EGFR* gene [2]. However, not all tumors have actionable genomic alterations and/or available matching targeted therapies. For example, majority of pancreatic cancers that harbor activating *KRAS* mutations have no effective targeted therapies [3]. Therefore, despite the promise of targeted therapies, chemotherapy still remains the most widely used standard of treatment for both advanced, metastatic cancer patients, and in the adjuvant setting.

While the use of predictive biomarkers in personalizing targeted cancer therapies is common, it is not yet a standard approach for chemotherapy. This is primarily due to the lack of compelling evidence from biomarker-driven studies to support their clinical utility [4–6]. Therefore, there is a clear need to comprehensively curate and evaluate literature-based datasets within an evidence framework to determine the quantity and quality of evidence supporting or refuting the clinical utility of these biomarkers. Such evidence frameworks currently exist to determine the clinical utility of predictive biomarkers for targeted therapies [7–9]. However, to our knowledge, this approach has not been used to assess the clinical utility of chemopredictive biomarkers. Our goal was to perform an exhaustive literature review to assess the overall levels of evidence supporting the clinical utility of a shortlist of chemopredictive biomarkers, using an evidence framework that is based on widely accepted guidelines. We hope this work can serve as a reference to evaluate future predictive biomarkers published in the field.

Numerous studies have shown that the expression of DNA repair genes like ERCC1, β-tubulins or topoisomerases can predict response to platinum, taxanes and other cytotoxic agents respectively [10–12]. We identified 7 biomarkers that have been evaluated in a number of tumor types (Table 1) for their role in predicting response to commonly approved chemotherapy drugs in the first-line, advanced, metastatic and adjuvant treatment settings. These biomarker therapy combinations are ERCC1–platinum drugs, RRM1–gemcitabine, TUBB3–taxanes, TYMS–5-fluorocuracil (5-FU)/Capecitabine, MGMT–temozolomide, TOP1–irinotecan/topotecan and TOP2A–anthracyclines. Several CLIA and/or CAP certified molecular diagnostic assays measure expression levels of these biomarkers in cancer tissue to determine their sensitivity to chemotherapy drugs. Therefore, we specifically examined how gene or protein expression changes in these biomarkers affect sensitivity or resistance to the associated chemotherapy agents.

**Table 1:** Summary of data for each biomarker-drug combination

Biomarker – Drug combination	# of studies screened (# of studies curated)	Most common cancer studied (# of studies)	Other cancers studied (# of studies, if ≥ 2)	Overall Evidence
ERCC1 – Platinum agents	266 (85)	Non-small cell lung cancer (43)	Ovarian cancer (10), Esophageal cancer (5), Small-cell lung cancer (4), Squamous cell head and neck cancers (HNSCC) (3), Colorectal cancer (3), Pancreatic cancer (2), Bladder cancer (2)	Consistent evidence from levels I-IV retrospective studies
MGMT – Temozolomide	366 (55)	Gliomas (25)	Pituitary tumors (9), Melanoma (6), Neuroendocrine tumors (2)	Modest evidence from levels III-IV studies
RRM1 – Gemcitabine	131 (55)	Non-small cell lung cancer (33)	Pancreatic cancer (7), Breast cancer (2)	Consistent evidence from levels I-IV retrospective studies
TS – 5-fluorouracil (5-FU), Capecitabine	617 (55)	Colorectal cancer (27)	Gastric cancer (13), esophageal cancer (5), Hepatocellular cancer (2), Pancreatic cancer (2)	Modest evidence from levels III-IV studies
TUBB3 – Taxanes	61 (40)	Non-small cell lung cancer (14)	Breast cancer (9), Gastric cancer (7), Ovarian cancer (3) Melanoma (2)	Modest evidence from levels III-IV studies
TOPO1 – Irinotecan, Topotecan	50 (11)	Colorectal cancer (5)		Weak evidence from few level III and IV studies
TOP2A – Anthracyclines	62 (17)	Breast cancer (13)	Hepatocellular carcinoma (2)	Weak evidence from few level III and IV studies

*Total number of studies screened and curated based on our inclusion criteria; the most commonly represented cancers for each biomarker-drug combination and the overall evidence supporting the predictive effect of each biomarker*.

**Figure 1:**
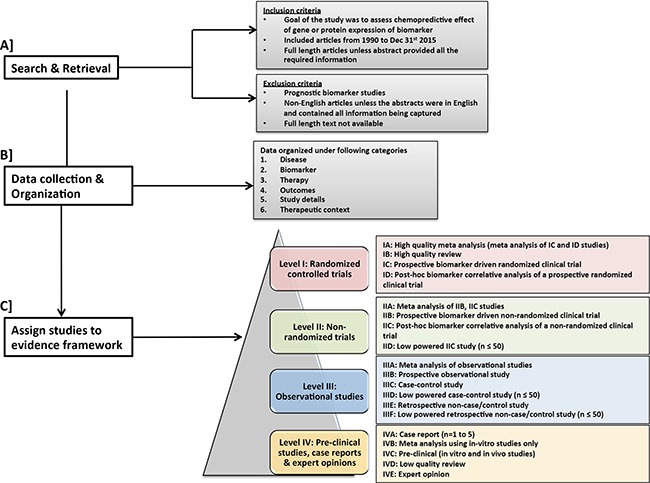
Workflow for search and retrieval and curation **A**. Search & retrieval and selection criteria for studies **B**. data collection and organization and **C**. Assignment of studies in the proposed evidence framework.

We conducted a comprehensive literature review from 1990-2015 on the predictive effect of gene or protein expression of these 7 biomarkers and associated chemotherapy drugs. The information from each study was systematically organized to minimize bias and maximize retrieval of relevant information in order to create a gold standard dataset (data available in supplementary tables). The level of evidence for each study was evaluated within an evidence framework which was adapted from widely accepted guidelines [13–15] Figure 1C. This dataset can inform future automation of information extraction through Natural Language Processing (NLP) approaches. Our results highlight a general need for more and higher quality level I evidence supporting clinical utility of chemopredictive biomarkers. Such an approach can help researchers and clinicians evaluate the clinical utility of chemopredictive biomarkers, thus enabling design of clinical trials and decision-making for patient care.

## METHODS

Figure 1 shows the overall workflow used to evaluate and summarize the evidence supporting the use of the 7 chemopredictive biomarkers for personalizing cancer treatment

### Search and retrieval

We searched PubTator [16] for the following keyword combination: gene/protein name, the term “expression” and “drug name”; for example “TUBB3 expression and taxanes”. Studies published between 1/1/1990 – 12/31/2015 that focused on predictive biomarkers whose gene or protein expression influenced response to chemotherapy were shortlisted for manual curation, Figure 1A. Articles that primarily focused on prognostic biomarkers were excluded. Non-English articles were excluded unless the abstracts contained all the necessary information to populate the framework. Important citations from the selected articles were also curated.

### Data collection

The following data elements were collected from each article and organized using controlled vocabulary wherever possible, Figure 1B:

- Disease – type of cancer and stage (early, advanced, metastatic, etc.)
- Biomarker – biomarker name, assay type used, other genes being studied, over and/or under expression status. The expression status is defined by the study and can be based on comparisons with all tumor samples, adjacent normal samples, or normal samples from unaffected individuals.
- Therapy – combinations of chemotherapy drugs that the patient/s was/were given and the therapy setting (first line, second line, adjuvant, etc.).
- Outcomes – outcome measures including, but not limited to, progression-free survival (PFS), disease-free survival, overall survival (OS), tumor response rate and tumor recurrence.
- Study details – model system being studied, for example cell line, animal models or human. For human studies we collected the inclusion, exclusion criteria and sample size, if available. Study metadata such as journal name, year of publication and publication source were also collected.
- Therapeutic context – predictive effect of biomarker expression on therapy outcome was interpreted from the results of the study.

### Biocuration

Expert curation and analysis was conducted by a multi-disciplinary group including: oncologists, molecular biologists, translational researchers, a surgeon, biocurators, bioinformaticians and biostatisticians.

### Predictive effect of biomarker

The predictive effect of the biomarker was determined from the results and conclusions reported in each study, captured under the following categories:

- Benefit: Over or under expression of the biomarker predicted sensitivity to therapy
- No Benefit: Over or under expression of the biomarker did not predict response to therapy
- Not Assessable: Results of the study were inconclusive for over and/or under expression of the biomarker.

### Evidence framework

Evidence levels broadly ranging from I-IV were assigned to each publication based on its study design Figure 1C, adapted from widely accepted guidelines [13–15]. Randomized clinical trials were assigned the highest evidence level I, followed by level II evidence for non-randomized trials, level III evidence for observational studies and lowest evidence level IV for pre-clinical studies, expert opinions and case studies. Within each evidence level, we incorporated sub-levels of evidence based on additional characteristics of study type, including prospective and retrospective analysis and sample size. Meta-analyses were usually assigned the highest sub-level evidence, since they integrate all the available evidence pertaining to a scientific question of interest and quantitatively summarize the results [17]. Depending on the type of studies (e.g. randomized, non-randomized, etc.) included in the meta-analyses, they were categorized under the corresponding evidence levels I-IV. Furthermore, within each evidence category ranging from I-III, prospective studies were ranked higher than retrospective studies. Systematic reviews were carefully selected based on stringent study inclusion criteria and were assigned high evidence level (IB). The term “high quality” meta-analysis or review in the figure refers to these stringent criteria, not to journal impact factor. Expert opinions that had a built-in rigorous meta-analysis were assigned to level I. Reviews including different study types and lacking stringent patient and/or study inclusion criteria were assigned a lower evidence level (IVD). Each study was considered individually for evidence assignment and no effort was made to combine evidence across studies using meta-analysis approaches.

## RESULTS

**Figure 2:**
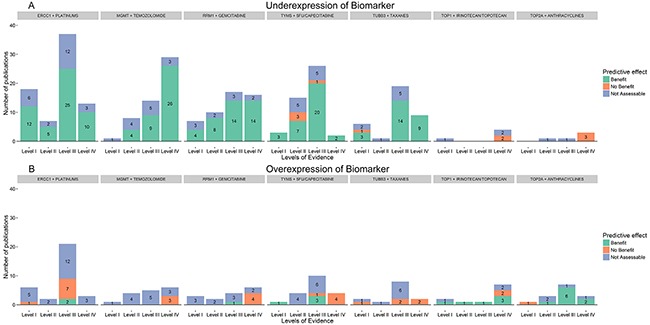
Overall evidence supporting the clinical utility of chemopredictive biomarkers Overall evidence associated with the predictive effect of biomarker expression on response to corresponding chemotherapy drugs defined in terms of benefit, no benefit or not assessable. Studies where chemotherapy response was not assessable for both, over and under expression of biomarkers have been represented twice, in the over and under expression section. For example, in the RRM1-Gemcitabine plot, a total of six level III studies showed that gemcitabine response is not assessable for both over and under expression of RRM1 and the same six studies are plotted in both over and under expression histograms (blue).

Results from the curation of the 7 chemopredictive biomarker-drug combinations are summarized in Table 1, presenting the total number of studies screened and shortlisted based on our inclusion criteria; the most commonly represented cancers for each biomarker-drug combination and a summary of the overall evidence supporting the predictive effect of each biomarker. Figure 2 shows a breakdown of the number of studies in each evidence level demonstrating the predictive effect of biomarker expression on response to corresponding chemotherapy drugs defined in terms of benefit, no benefit or not assessable. Further details on therapy setting, sample size, outcomes and other study details are available in the supplementary tables.

### Excision repair cross-complementing group 1 (ERCC1) - platinum drugs

Pre-clinical studies have suggested that underexpression of ERCC1 sensitizes cancer cells to platinum agents whereas overexpression induces resistance [18, 19]. 70% of the studies in our dataset confirm this hypothesis, where the majority of the evidence is from levels I-III retrospective studies. The predictive effect of ERCC1 underexpression on response to platinum agents has been widely studied in NSCLC (Table 1). Out of the two level IC clinical trials in our dataset, one study had no benefit for PFS. In this study, an internal control suggested the possibility of biased randomization, thereby confounding the result, and therefore we have determined the results of this study to be not assessable [20]. A second level IC study confirmed a modest response rate advantage (the primary endpoint), while failing to confirm PFS benefit (secondary endpoint). While we have counted this study as supporting the biomarker hypothesis, a study evaluating the clinical benefit endpoint as primary would be very helpful in determining the clinical significance of this result [21]. Two level III studies showed contradictory evidence in advanced NSCLC and metastatic pancreatic cancer patients where overexpression of ERCC1 showed benefit from platinum based chemotherapy [22, 23]. 22 studies in our dataset had results that were not assessable due to various reasons including different methods of biomarker quantification, disease type, stage and small sample size [24–28] (Supplementary Table S1). While the evidence supporting the chemosensitizing effect of ERCC1 underexpression in response to platinum agents was somewhat consistent across all retrospective studies in NSCLC, the evidence for other cancer types was often inconclusive or contradictory. The overall evidence for other cancer types was mainly from low-level III-IV studies [29–33]. Moreover, the predictive effect of immunostaining for ERCC1 protein has been difficult to confirm in validation studies. This can be attributed to discrepancies in the performance of antibody batches over the years and also a lack of understanding of the heterogeneous expression and function of different ERCC1 protein isoforms [34].

### O6-methylguanine DNA methyltransferase (MGMT) - temozolomide

Underexpression of MGMT is thought to sensitize tumor cells to temozolomide (TMZ)-based therapies whereas overexpression induces resistance [35–38]. This hypothesis was widely tested and confirmed in gliomas and pituitary tumors (Table 1, Supplementary Table S2). We found that the overall evidence supporting the chemosensitizing effect of MGMT underexpression on TMZ response is mainly from level IV pre-clinical studies and a few level II-III retrospective studies (Figure 2). The results from 13 studies, including a high-level ID retrospective biomarker study were not assessable [39–42]. In addition to gene/protein expression, we also found several studies that evaluated the predictive effect of MGMT promoter methylation on TMZ response, including the landmark study by Hegi et.al [43] that drives the current clinical use of MGMT as a predictive biomarker for TMZ response in glioblastoma multiforme. Such studies were not included in our dataset since they did not meet our inclusion criteria, which focused only on predictive biomarkers whose gene/protein expression influenced response to chemotherapy. In studies that tested the predictive effect of both MGMT expression and promoter methylation on TMZ response, there was often poor correlation between the two, which resulted in inconclusive results [44].

### Ribonucleotide reductase subunit M1 (RRM1) - gemcitabine

Pre-clinical studies have shown that RRM1 underexpression is associated with benefit of gemcitabine-based therapy whereas overexpression is associated with resistance [45–47]. This trend is consistent in 80% of the studies across all evidence levels I-IV. The predictive effect of RRM1 underexpression on benefit of response to gemcitabine was mainly studied in NSCLCs and confirmed by a level IC clinical trial [20]. In pancreatic cancers, the overall evidence supporting the predictive effect of RRM1 was equivocal and investigated only in levels III-IV studies [48–55].

A number of NSCLC studies analyzed the combined predictive effect of both RRM1 and ERCC1 expression, perhaps because treatment for NSCLCs usually includes gemcitabine in combination with platinum drugs. While a level IIA multi-trial NSCLC study confirmed that patients who received personalized first-line therapy based on their RRM1 and ERCC1 gene expression status had better survival than patients on standard therapy [56], a level IIIF study of a three drug concurrent regimen [57] produced results which were contradictory to previous studies, in that responders had low gene expression levels ERCC1 as expected but high levels of RRM1. It can be difficult to determine single marker outcomes from such multi-marker studies.

### Thymidylate synthase (TYMS)-5-fluorouracil/capecitabine

Pre-clinical studies have demonstrated that underexpression of TYMS predicts benefit of response to 5-fluorouracil (5-FU)-based therapies whereas overexpression predicts no benefit [58–64]. Our dataset also included studies that tested TYMS expression and response to 5-FU's prodrug, Capecitabine [10, 64–68]. We found that evidence supporting the chemosensitizing effect of TYMS under expression on 5-FU/Capecitabine is mainly from levels II-III retrospective biomarker analysis of clinical studies (Figure 2). This hypothesis was widely tested and confirmed in gastrointestinal cancers especially colorectal cancers (Table 1, Supplementary Table S4). However, the only level IC clinical trial in our dataset showed contradictory evidence where metastatic colorectal cancer patients overexpressing TYMS had a trend towards better overall survival than the underexpressing cases [69]. The chemosensitizing effect of TYMS underexpression was not assessable in 11 studies whereas 3 studies showed no benefit of TYMS underexpression on 5-FU therapy. Further prospective studies in large well-defined patient populations are necessary to determine the clinical utility of this biomarker. Moreover, ~27 studies in our dataset examined the predictive effect of other biomarkers like TP, DPD and ERCC1 in addition to TYMS in response to 5-FU based therapies. This makes it difficult to determine the predictive effect of TYMS alone on 5-FU response [70].

### Class III beta-tubulin (TUBB3)-taxanes

Pre-clinical studies have suggested that underexpression of TUBB3 predicts sensitivity to taxanes whereas overexpression predicts resistance in breast cancer, NSCLC and gastric cancers [71–73]. This hypothesis was mainly supported by 11 level IV pre-clinical studies and 14 level III retrospective studies (Table 1, Figure 2). We did not find any evidence from prospective biomarker driven trials to support the predictive effect of TUBB3 underexpression on response to taxanes. The results from 9 studies that evaluated taxane response in patients under/over expressing TUBB3 were not assessable [74–82]. In a level ID breast cancer study [76], patients overexpressing TUBB3 had a higher probability of response to docetaxel but the predictive effect of TUBB3 underexpression was not assessable. This unusual predictive effect of TUBB3 expression has also been reported in patient populations that received taxanes in an adjuvant or advanced disease setting [83].

### Type I topoisomerase (TOP1)-irinotecan/topotecan

Studies on gastrointestinal cancers have suggested that overexpression of TOP1 predicts benefit of camptothecin-based therapies [84–88]. However, the evidence associated with the predictive effect of TOP1 expression status and therapy outcome was mostly of low-level and studies often reported inconclusive results [89–91] (Figure 2). A level ID post-hoc biomarker correlative analysis of the FOCUS trial showed an overall survival benefit in a subset of colorectal cancer patients with moderate/high TOP1 levels measured by immunohistochemistry [92]. However, another similar level ID study conducted by the Dutch Colorectal Cancer Group showed no association between TOP1 expression and survival benefit [93]. Moreover, Meisenberg et al. reported an important correlation between TOP1 and TDP1, suggesting the possible role of other predictive markers in irinotecan/topotecan sensitivity [94].

### Topoisomerase (DNA) II alpha (TOP2A) - anthracyclines

The TOP2A gene is overexpressed in several cancer types and is hypothesized to predict sensitivity to anthracycline-based therapies [95, 96]. However, our only high evidence study (level ID) on hepatocellular carcinoma patients showed no benefit of TOP2A overexpression on anthracycline based therapies [97]. 71% of the studies in our dataset showed levels II-IV evidence supporting this hypothesis, where either overexpression of TOP2A was associated with benefit or underexpression was associated with no benefit from anthracycline-based therapy (Figure 2, Supplementary Table S7). Since anthracyclines are frequently used in breast cancer treatments, we found this disease to be most widely represented in our dataset, especially in HER2+ breast cancer. However, we did not find any high evidence level I studies on TOP2A overexpression as a predictor of response to anthracyclines in this review and analysis.

## DISCUSSION

Herein, we provided a high level quantitative perspective on the amount and quality of evidence supporting or contradicting the clinical utility of chemopredictive biomarkers. In our evaluation, we found that biomarker-driven prospective clinical trials (levels I-II) for these protein-drug pairs were few and often reported findings that were inconclusive or contradictory. We found somewhat consistent evidence from several retrospective biomarker analyses across levels I-III in NSCLC supporting the chemosensitizing effect of both ERCC1 and RRM1 underexpression in response to platinum and gemcitabine based treatments respectively. The evidence supporting the predictive role of gene/protein expression of TYMS–5FU/Capecitabine in colorectal cancers, TUBB3-taxanes in NSCLC and MGMT–temozolomide in brain tumors was modest and mainly from level III observational studies and level IV pre-clinical studies. There was sparse evidence from level III retrospective studies and level IV pre-clinical studies supporting the chemosensitizing effect of TOP1 and TOP2A overexpression in response to camptothecin-based therapies and anthracyclines in gastrointestinal and breast cancers respectively. Other studies that assessed promoter methylation in MGMT and amplification in TOP1 and TOP2A were not considered in this assessment. Our analysis highlights the need for more well-designed and higher quality level I evidence studies for the 7 chemopredictive biomarker – drug pairs.

Standardizing and organizing relevant information from different biomarker studies presents several challenges 1) author preferences in presenting the information 2) author bias resulting in discrepancies between the summary statements and the actual evidence 3) diversity of methods used to quantitatively measure biomarker levels 4) inconsistency in results and conclusions from different assays, statistical tests or for different endpoints, and 5) inconsistency in clinical information across tumor types. Moreover, since chemotherapy drugs are often given in combination, it may be difficult to determine the predictive effect of a single biomarker-drug combination.

The work summarized herein focuses the effort on a manageable number of studies, which need to be reviewed in greater detail, making such a task feasible. Level I evidence outweighs lower levels of evidence but within the level I evidence framework all studies cannot be considered equal. More detailed curation of such studies is required to rank them based on the strength of evidence they provide. Critical factors to assess within the level I evidence category include study population, endpoints, and appropriate controls; amount of missing data; assay validation; sample collection and processing; statistical and clinical significance of the results, and confounders. Evaluation of these criteria may help to resolve conflicts between studies if one provides stronger evidence than another. Limitations of the current level I studies hamper our ability to draw firm conclusions about the current clinical applicability of these biomarkers.

As more data emerges on novel “actionable” pathways in cancers, this evidence framework will need further development as outlined above to assess clinical actionability. The framework can be applied to enhance the value of the increasing volume of retrospective data collection. However, the framework will likely highlight the need for additional prospective studies and help guide their design. These critical future steps will allow the cancer precision medicine community to collectively evaluate and accept predictive biomarkers for cancer therapy.

## SUPPLEMENTARY MATERIALS TABLES
















