# Supplementary material for: Quantification and expert evaluation of evidence for chemopredictive biomarkers to personalize cancer treatment
Source: Oncotarget. 2016 Nov 24;8(23):37923–34. doi: 10.18632/oncotarget.13544 (PMC5514962; doi:10.18632/oncotarget.13544)
Supplement: Supplementary file 1 [file oncotarget-08-37923-s001.pdf]

Quantification and expert evaluation of evidence for chemopredictive biomarkers to personalize cancer treatment

SUPPLEMENTARY TABLES

Supplementary Table S1: Evidence for ERCC1 and platinum drugs.

See Supplementary File 1

Supplementary Table S2: Evidence for MGMT and temozolomide.

See Supplementary File 2

Supplementary Table S3: Evidence for RRM1 and gemcitabine.

See Supplementary File 3

Supplementary Table S4: Evidence for TYMS and 5-FU/capecitabine.

See Supplemenatry File 4

Supplementary Table S5: Evidence for TUBB3 and taxanes.

See Supplementary File 5

Supplementary Table S6: Evidence for TOP1 and irinotecan/topotecan.

See Supplementary File 6

Supplementary Table S7: Evidence for TOP2A and anthracyclines.

See Supplementary File 7
